# Effect of local somatosensory stimulus on postural sway during sit-to-stand movement in the elderly

**DOI:** 10.1186/s12891-021-04609-7

**Published:** 2021-11-12

**Authors:** Peter Annor, Kiyoung Kwak, Huigyun Kim, Dongwook Kim

**Affiliations:** 1 Department of Healthcare Engineering, Graduate School, Jeonbuk National University, 567, Baekje-daero, Deokjin-gu, Jeonju-si, Jeollabuk-do Republic of Korea; 2 Division of Biomedical Engineering, College of Engineering, Jeonbuk National University, 567, Baekje-daero, Deokjin-gu, Jeonju-si, Jeollabuk-do Republic of Korea; 3 Research center for Healthcare & Welfare Instrument for the Elderly, Jeonbuk National University, 567, Baekje-daero, Deokjin-gu, Jeonju-si, Jeollabuk-do Republic of Korea

**Keywords:** Posture control, Elderly, Local tendon vibration (LTV), Sit to stand movement (STS), Center of pressure (COP), Center of mass (COM)

## Abstract

**Background:**

Sit-to-stand (STS) is a complex movement that requires successful postural control. Aging is a normal part of human life that leads to weakness of sensory capabilities, resulting in diminished postural control. Therefore, STS movement is a challenging task for the elderly. Local tendon vibration (LTV) can be utilized to assist STS of the elderly by improving postural control. Many studies have revealed that the LTV has various physiological positive effect. However, previous studies did not consider subjects’ individual difference for properties of applied LTV. Also, there are almost no studies to assist and to improve elder’s STS movement. Thus, the purpose of this study was to examine the influence of lower limb LTV on postural sway during STS in the elderly, and to examine whether a specific vibration frequency can increase postural control in the elderly.

**Results:**

The common characteristic differences between the elderly and younger population during STS movement were analyzed. In addition, the effect of vibration on the center of mass (COM) and the center of pressure (COP) variable responses in young adults and the elderly were investigated. As a result, the elderly exhibit larger COP sway area and higher COP mediolateral (ML) displacement than the young adults. In addition, the elderly generally have lower COM velocities in all directions compared to the young adults. It was found that COP and COM related to postural stability are affected when LTV of the 180 Hz, 190 Hz and 250 Hz is applied to the elderly. Particularly, the 190 Hz vibration induced significant reduction in COP sway area and COP ML displacement.

**Conclusions:**

These results mean that the LTV contributes to stability of elders’ STS movement by reducing postural sway. Furthermore, a reduction of postural sway depends on frequency of the LTV. These findings suggest that individual response to characteristics of vibration must be considered, and imply that the LTV can be used as rehabilitation therapy to improve postural control in the elderly, and utilized in motion assistive devices to deliver apt vibration frequencies.

**Trial registration:**

CRIS, KCT0005434, Registered 25 September 2020, Retrospectively registered, https://cris.nih.go.kr/cris/index/index.do

## Background

Sit-to-stand (STS) movement is a common activity of daily life [1]. Rising from the seated position is a complex dynamic activity. It involves postural control during momentum transfer from a 3-points stable base, the sitting location, to a 2-points base, the standing location [1]. Persons with a range of motor disabilities have problems or incapacity to stand up [1]. During STS movement, the weight of the body must be shifted carefully inside the base of support to prevent falls [2]. Thus, the ability to control the center of mass (COM) is very important for the successful STS movement. When the COM is propelled too fast in the forward direction, a fall is likely to occur [3]. In addition, if the center of pressure (COP) is displaced more in the medio-lateral (ML) direction, the likelihood of a fall to the side increases [4]. The ability to effectively control ML postural stability is linked to many elderly falls [2–4].

Aging is a natural process that every person experiences at some point in time. It is accompanied by several changes in the body, such as a decrease in the range of motion and muscle strength [5] and sometimes a loss of senses. The ability to perform STS movements depends on good postural control.

Aging creates a significant deficit in the postural control system. This causes an increase in the displacement and velocity of COP sway during static and dynamic activities [6].

Aging weakens the neurophysiological abilities of the body, resulting in loss of stability during STS movement. Some of the causes of poor postural control in older adults are changes in muscle activation pattern, loss of sensory system and decrease in muscle strength [6, 7]. Any of these is enough to cause a fall in the elderly during motion. Postural instability contributes greatly to falls during STS movement, especially in old persons.

According to WHO reports published in 2018, falls are the second leading cause of accidental injury and premature death [8, 9]. Every year, approximately 646,000 persons die from falls worldwide, out of which adults older than 65 years suffer the most number of fatal falls, with an incidence that increases with age [9, 10]. The majority of falls occur during daily activities, such as STS movement and walking [1, 11].

Postural control is maintained by several modalities that send information to the central nervous system. When there is an imbalance, a motor response is sent to restore stability. However, in the elderly, this response is usually delayed, and falls can frequently occur during sway. One way researchers have attempted to increase the motor response in the elderly is through local tendon vibration (LTV).

Some researchers have studied the neurological and physiological effects of LTV stimulation. LTV stimulation is the application of vibration to the muscle belly or tendon. Studies have shown that it can improve muscle power, muscle strength and postural control during dynamic activities [12].

Many researchers have attempted to apply varying frequencies and amplitudes of vibration on the lower extremities to study its effect on muscle strength and performance in various participants. For STS movement, Kurokawa et al. [13] studied postural response upon applying vibration simulation at a frequency of 100 Hz to the Achilles tendon during STS movement. The results proved the effectiveness of vibration stimulation on sensory information transmission. After the vibration was applied, there was an immediate response resulting in the body propelling forward during the STS movement. Their study revealed that LTV influences postural balance. However, there are some limitations. Analysis parameters were the leaning forward and backward, and the subjects were in their 20s, and one frequency was used. In addition, there are few studies on STS motion in the elderly, and there are no studies on STS motion assistance in the elderly using LTV.

Thus, to assist and improve postural control of STS movement in the elderly, the purpose of present study was to examine features of elderly STS movement through comparing with young adults, and to investigate effects of LTV on COP and COM during STS movement using various frequencies.

## Methods

### Subjects

This study was conducted on 15 young adults (age 25.5 ± 1.5 years old, height: 173.2 ± 2.6, weight: 72.4 ± 4.4 kg) and 10 elderly subjects (age: 76.0 ± 1.7 years old, height: 166.7 ± 3.7 cm, weight: 68.0 ± 5.5 kg). All the participants were healthy at the time of the experiment and could independently perform the STS activity. Before the experiment, the participants were informed about the requirements of the study, the procedure involved, and they then signed an informed consent document. This experiment was approved by the Institutional Review Board of the Jeonbuk National University (IRB File No. JBNU 2017–03–011-001).

### Local tendon vibration

The LTV was applied at four main points (biceps femoris tendon, Achilles tendon, quadriceps femoris tendon, and tibialis anterior tendon) during the STS movement at 180 Hz, 190 Hz, and 250 Hz. Linear actuators (DMJBRN0934AA, Samsung Electro-Mechanics Co., Ltd., Korea) were firmly attached to the target points, and a function generator (AFG-2125, Good Will Instrument Co., Ltd., Taiwan) was used to regulate the vibration frequency. The LTV stimulations was set in a total of four as follows: None stimulation (without the LTV), 180 Hz, 190 Hz, and 250 Hz.

### Protocol

To measure the STS movement, the participants’ starting position was standardized. Each participant wore shoes and was seated on an armless backless platform, which had one force plate on the seat area and two force plates for each leg. The subjects placed their feet on each force plate with their feet 10–15 cm apart. Each participant’s ankle was placed at approximately 10 degree of dorsiflexion, and the knee angle was approximately 100–105 degrees of flexion. The participants were then instructed to stand at their usual self-paced, comfortable speed. After the command “ready” was given, the STS movement was initiated when a buzzer was sounded. After approximately 5 sec of the entire STS process, the participants were instructed to sit normally. Three trials were performed for every vibration frequency.

### STS movement capture

**Fig. 1:**
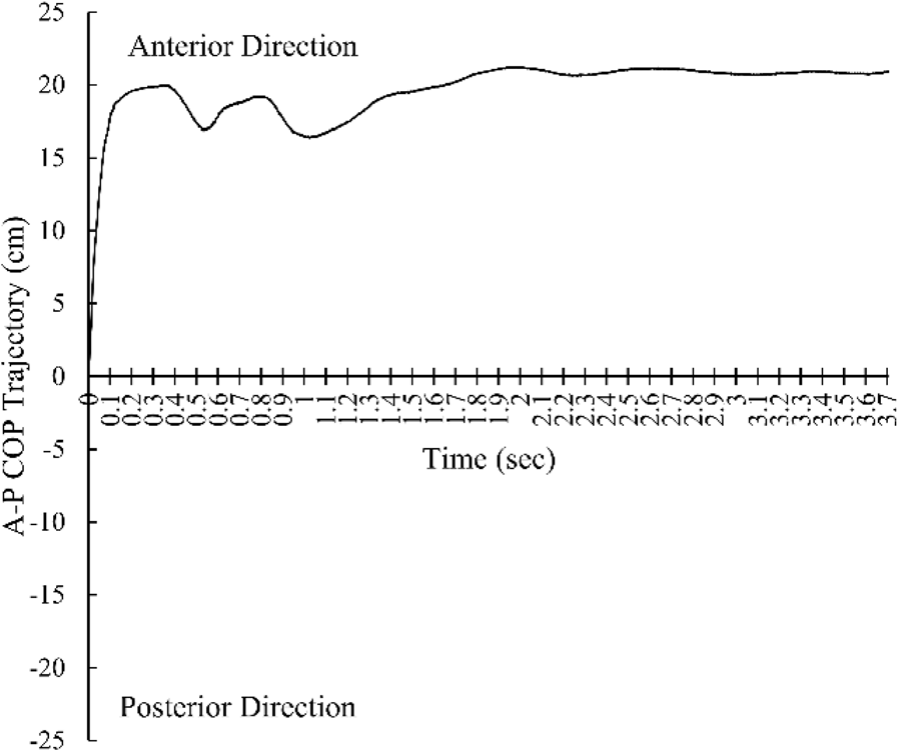
COP time-trajectory in anterior-posterior direction. Positive value indicates anterior direction. Negative value indicates posterior direction

**Fig. 2:**
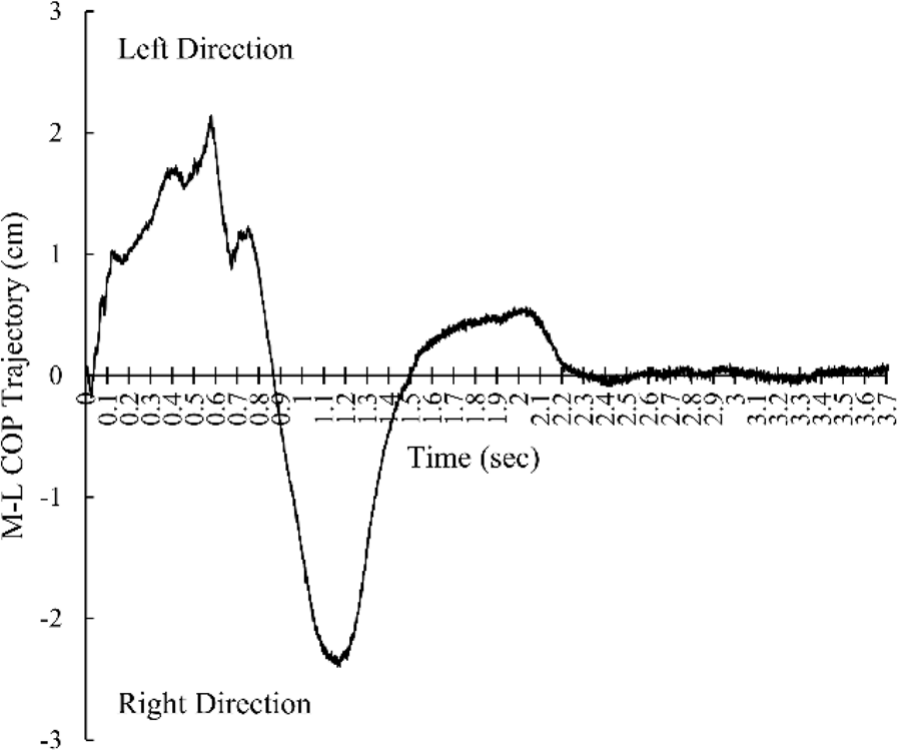
COP time-trajectory in medio-lateral direction. Positive value indicates left direction. Negative value indicates right direction.

**Fig. 3:**
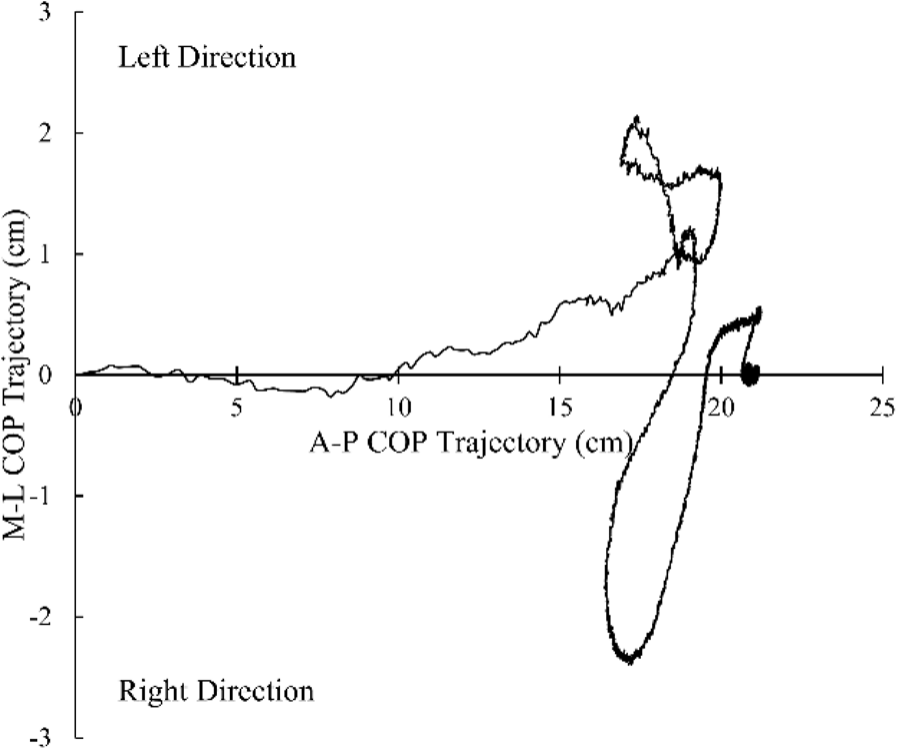
COP time-trajectory in all direction. Positive Y axis indicates left direction. Negative Y axis indicates right direction. Positive X axis indicates anterior direction

To capture the STS movement, 3-dimensional marker-based optical motion capture system which considered as the standard method of movement analysis [14] was used. A total of 11 active infrared emitting diodes were attached to each main joint as stated in the Halen-Hays marker set. A total of 3 force plates (Bertec Co. Ltd., USA) were used to calculate the ground reaction force during the STS movement. A total of three position sensors (Optotrak Certus, Northern Digital Inc., Canada) were used to obtain the infrared light from the markers. The First Principle software (Northern Digital Inc., Canada) was capture marker data and COP values during the STS movement. Figures 1, 2 and 3 represent COP time-trajectory in anterior-posterior (AP) direction, ML direction and combined AP-ML direction, respectively.

### COP and COM measurements

The STS movement was divided into phases according to Pavao et al. [15]. The preparation phase (PP), including the beginning of anterior trunk flexion to maximum flexion, when the body starts the seat-off [15]. The rising phase (RP), from the seat-off (maximum anterior flexion of the trunk) to standing posture [15]. The stabilization phase (SP), which involves maintaining the body in a quasi-stationary position [15]. The total phase (TP) is that the beginning of the PP to end of the SP. For each of the four phases of the STS movement, the COP parameters measured are COP area, COP path, and COP displacement in both AP and the ML direction. The COM parameters measured included COM velocity in the AP, ML, and vertical (V) directions and COM displacement. The raw COP position data from the force plates were set to a starting point of 0, and the results were time normalized from 0 to 100. The COP variables were calculated according to Piao et al. [16] as shown in the formula below.

1 $$ COP\  Sway\ path\ length=\sum \limits_{i=1}^{n-1}\sqrt{{\left({X}_i-{X}_{i-1}\right)}^2+{\left({Y}_i-{Y}_{i-1}\right)}^2} $$

$$ COP\  Sway\ area=\sum \limits_{i=1}^{n-1}\sqrt{S_i\left({S}_i-{a}_i\right)\left({S}_i-{b}_i\right)\left({S}_i-{c}_i\right)} $$ (2).$$ {a}_i=\sqrt{{X_i}^2+{Y_i}^2} $$ (3)

4 $$ {b}_i=\sqrt{{X_{i+1}}^2+{Y_{i+1}}^2} $$

5 $$ {c}_i=\sqrt{{\left({X}_{i+1}-{X}_i\right)}^2+{\left({Y}_{i+1}-{Y}_i\right)}^2} $$

6 $$ {S}_i=\frac{a_i+{b}_i+{c}_i}{2} $$

7 $$ COP\  RMS\  AP=\sqrt{\frac{1}{n}\ast \sum \limits_{i=1}^{n-1}{\left({X}_i-{X}_{i-1}\right)}^2} $$

Where n is the total number of samples, *i* is the index of a current sample, *X* is the COP displacement in the AP direction, *Y* is the COP displacement in the ML direction. *a, b* and *c* are the lengths of the sides, *S* is the half of the triangle’s perimeter [16].

The COM values were obtained from the Software for interactive musculoskeletal Modeling SIMM 7.0 (MusculoGraphics, Inc., USA) for all three phases of STS and the calculations were performed based on the vector positions as shown below.

8 $$ COM\  displacement=\sum \limits_{i=1}^n\sqrt{{\left({X}_i-{X}_{i-1}\right)}^2+{\left({Y}_i-{Y}_{i-1}\right)}^2+{\left({Z}_i-{Z}_{i-1}\right)}^2} $$

9 $$ COM\  Velocity\ x=\frac{1}{n}\ast \sum \limits_{i=1}^n\left({V}_{Xi}\right) $$

10 $$ COM\  Velocity\ y=\frac{1}{n}\ast \sum \limits_{i=1}^n\left({V}_{Yi}\right) $$

11 $$ COM\  Velocity\ z=\frac{1}{n}\ast \sum \limits_{i=1}^n\left({V}_{Zi}\right) $$

Where n is the total number of samples, *i* is the index of a current sample, *X* is the COM displacement in the AP direction, *Y* is the COM displacement in the ML direction, V is the COM displacement in the vertical direction, *Vx, Vy* and *Vz* are the velocities in the AP, ML, and V directions, respectively.

### Statistical analysis

The collected data were analyzed using SPSS 25.0 (IBM Corp., USA). Descriptive statistics was used to compute the mean and standard deviation. The Kolmogorov-Smirnov test was performed to test for normality. The paired t-test was performed for comparisons between the cases with no vibration and those with 180 Hz, 190 Hz, and 250 Hz frequency vibrations for all the phases in the STS movement. An independent sample t-test was used to compare the features between young adults and the elderly. The statistical significance level was set at 0.05.

## Results

### General differences between young and elderly

#### COP area and COP path length

**Fig. 4:**
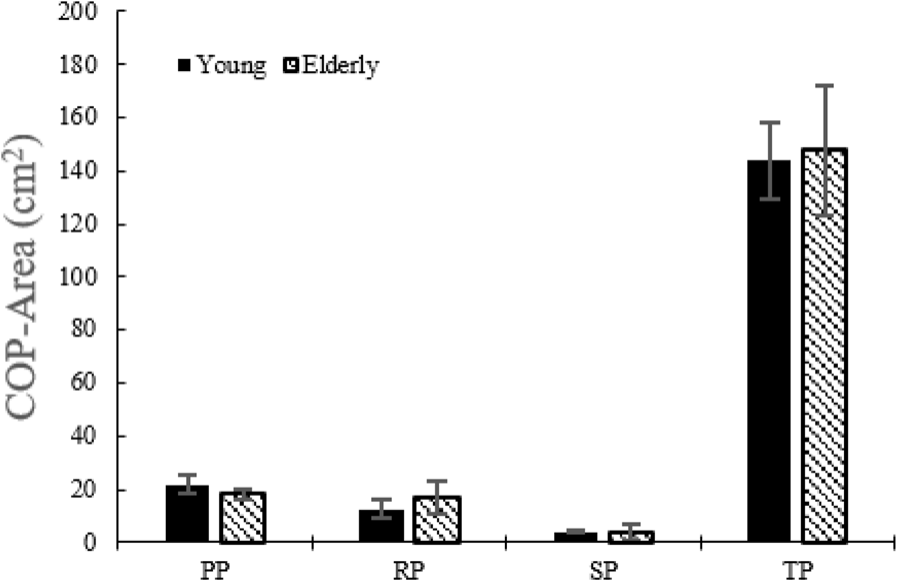
COP area without the LTV stimulation for young adults and the elderly

**Fig. 5:**
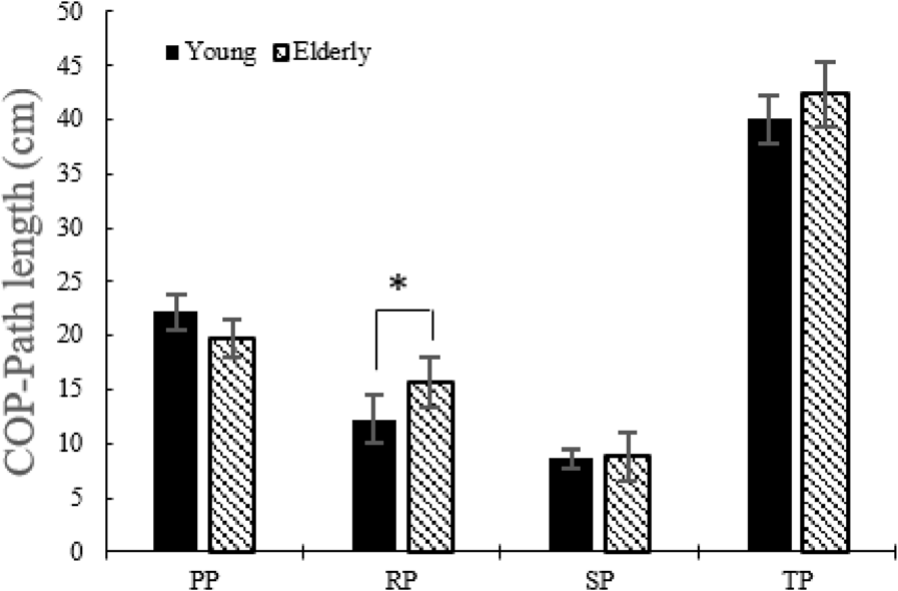
COP path length without the LTV stimulation for young adults and the elderly

Figure 4 shows no difference (*p* > 0.05) in the COP area between the young and the elderly during all phases. Elderly persons showed higher (*p* < 0.05) COP path length during the RP (Fig. 5).

Although there were no statistical significances, the elderly showed higher postural sway during the TP. Particularly, in the RP among sub-phases, higher postural sway was more prominent compared to the young adults.

#### COP displacement of COP RMS in AP and ML directions

**Fig. 6:**
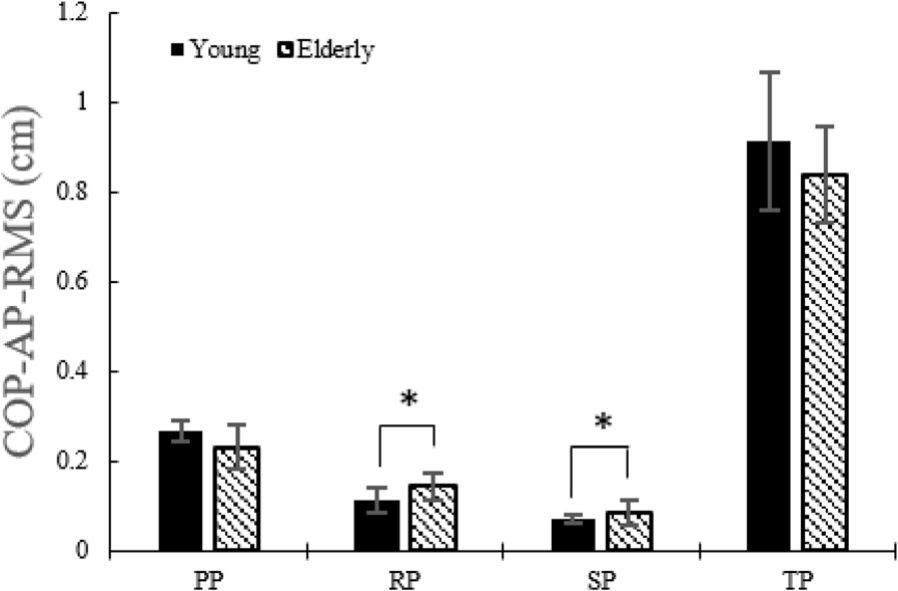
COP RMS in the AP direction without the LTV stimulation for young and the elderly

**Fig. 7:**
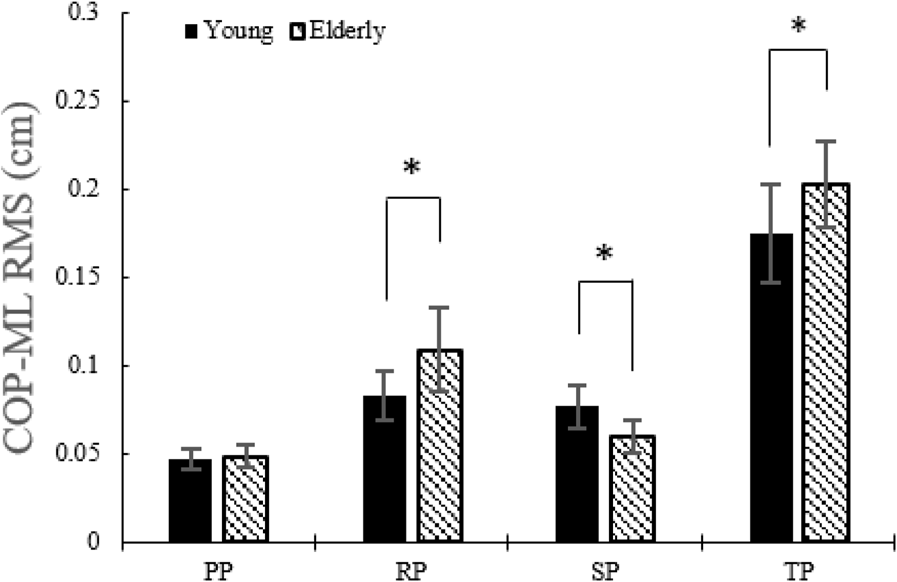
COP RMS in the ML direction without the LTV stimulation for young and the elderly

Figure 6 indicates that COP RMS in the AP direction was higher (*p* < 0.05) in the elderly than in the young during the RP and the SP. COP RMS in the ML direction was higher (*p* < 0.05) in elderly during the RP and the TP but reduced during the SP (Fig. 7).

While performing STS movement, the elderly showed higher postural sway in the ML direction. To examine with more details regarding the sub-phases, in the AP direction, higher postural sway appeared in both the RP and SP compared with the young adults. On the other hand, in the ML direction, higher postural sway appeared only in the RP.

#### COM displacement and COM velocity in AP direction and time duration

**Fig. 8:**
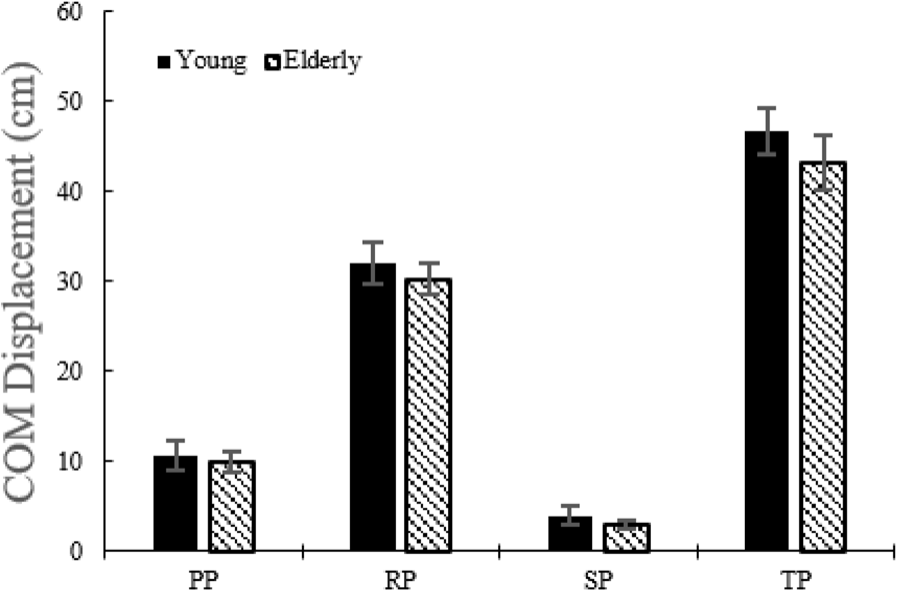
COM displacement without the LTV stimulation for young adults and the elderly

**Fig. 9:**
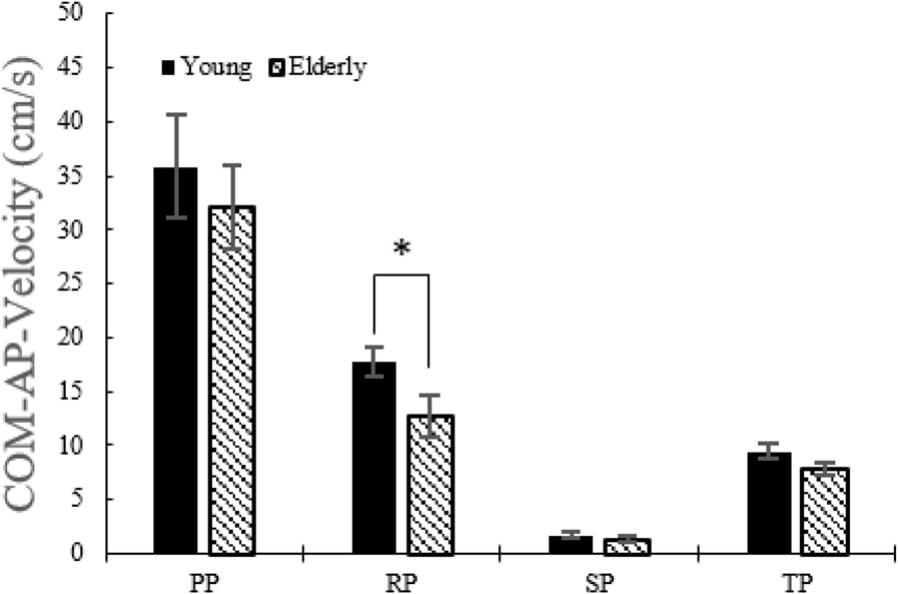
COM velocity in the AP direction without the LTV stimulation for young adults and the elderly

**Fig. 10:**
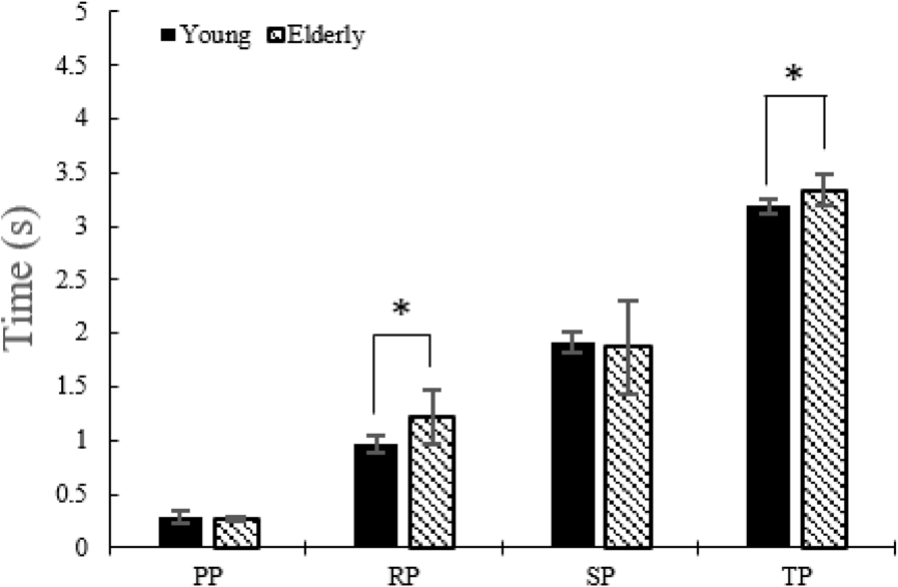
Time durations without the LTV stimulation for young adults and elderly

There was no difference in the COM displacements between the two groups (Fig. 8). During the RP, COM velocity in the AP direction in young adults is higher than in the elderly (Fig. 9). During the RP and the TP, time durations were lower in the case of young adults than in the elderly (Fig. 10).

Overall, the elderly move their weight slowly during STS. This means that momentum transfer is achieved slowly, requiring more time to complete STS and also causes more COP sway in the AP direction.

#### COM velocity along ML and V directions

**Fig. 11:**
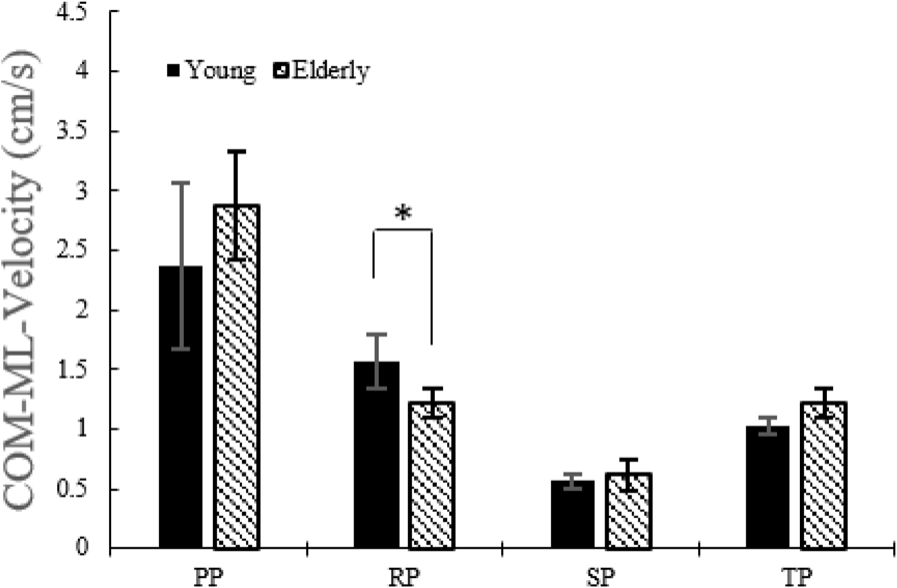
COM velocities in the ML direction without the LTV stimulation for young and the elderly

**Fig. 12:**
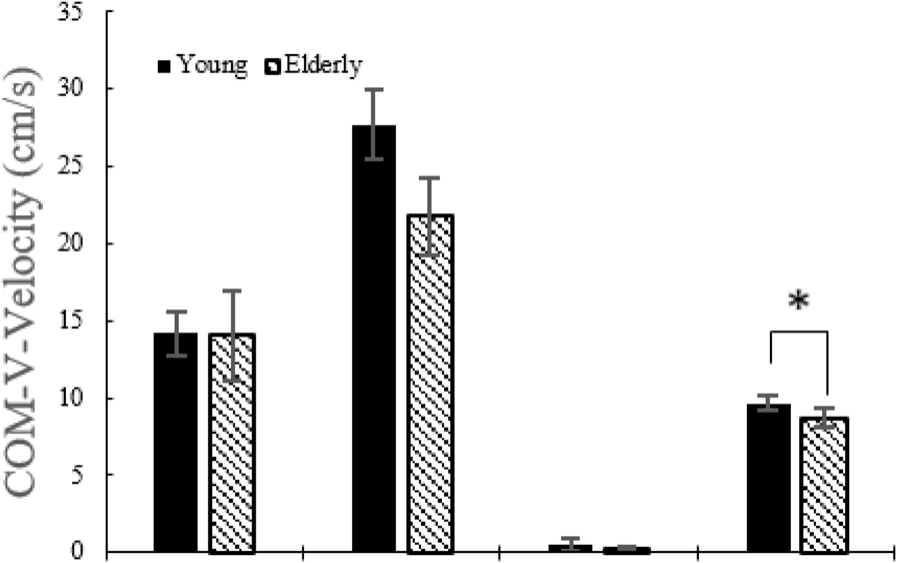
COM velocities in the V direction without the LTV stimulation for young and the elderly

Figure 11 showed that young adults have a higher COM ML velocity than the elderly during the RP. COM velocity V in young adults is higher than in the elderly during the TP (Fig. 12). Likewise the COM AP velocity, weight shift was achieved slowly at the RP in the ML direction. In addition, although statistical difference wasn’t discovered, same result appeared in the V direction. Consequently, the elderly show a noticeably lower postural control and weight shift ability in the RP.

### Change of COM and COP with local tendon vibration

#### COM displacement

The TP (Table 1) showed a COM displacement increase (*p* < 0.05) at 190 Hz and 250 Hz in young adults. The TP (Table 2) showed that 190 Hz and 250 Hz vibrations in the elderly reduce COM displacements (*p* < 0.05).

**Table 1:** COM displacement in young adults

STS phase	None stimulation	180 Hz	190 Hz	250 Hz
PP	10.65	10.84	11.15	11.07
	±1.64	±1.59	±1.50	±1.76
RP	32.00	32.90	32.85	33.18
	±2.33	**±**1.60	±1.35	±1.50
SP	4.03	3.44	3.37	3.46
	±1.01	±0.53	**±**0.53	**±**0.72
TP	46.66	47.15	47.50*	47.68*
	±2.56	±1.82	±1.79	±1.60

*: *p* < 0.05. None stimulation versus stimulation, mean ± standard deviation (cm)

**Table 2:** COM displacement in the elderly

STS phase	None stimulation	180 Hz	190 Hz	250 Hz
PP	9.94	10.26	9.29	9.62
	±1.21	±1.73	±1.62	±1.76
RP	30.30	30.53	31.05	30.84
	±1.81	**±**1.72	±1.83	±2.14
SP	3.02	2.70	2.67	2.24
	±0.55	±9.9	**±**0.53	**±**0.48
TP	43.25	43.47	43.00*	42.69*
	±3.02	±2.36	±2.73	±2.77

*: *p* < 0.05. None stimulation versus stimulation, mean ± standard deviation (cm)

In both groups, COM displacement decreased in the TP, which be attributed to decreased COM displacement in the SP. From this result, it can be seen that the vibrations of the 190 Hz and 250 Hz in particular contribute to the reduction of COM sway.

### COM velocity along AP direction

No significant difference was observed in any of the phases for young adults (Table 3), except in the velocity in the TP, which increased (*p* < 0.05) at 190 Hz. In the elderly (Table 4), the velocity in the TP increased (*p* < 0.05) at 180 Hz and 190 Hz.

**Table 3:** COM velocity along AP direction in young adults

STS phase	None stimulation	180 Hz	190 Hz	250 Hz
PP	35.88	37.02	37.73	37.70
	±4.75	±4.20	±4.34	±4.10
RP	17.75	7.56	12.72	17.46
	±1.39	**±**1.48	±1.25	±1.79
SP	1.68	1.63	1.67	1.73
	±0.30	**±**0.19	**±**0.27	**±**0.36
TP	9.46	9.87	10.09*	10.01
	±0.72	**±**0.64	±0.65	±0.67

*: *p* < 0.05. None stimulation versus stimulation, mean ± standard deviation (cm/s)

**Table 4:** COM velocity along AP direction in the elderly

STS phase	None stimulation	180 Hz	190 Hz	250 Hz
PP	32.13	32.71	33.56	34.67
	±3.84	±3.70	±4.32	±4.11
RP	12.77	12.10	12.72	12.09
	±1.93	**±**1.24	±1.04	±1.13
SP	1.33	1.27	1.27	1.01
	±0.28	**±**0.34	**±**0.30	**±**0.20
TP	7.88	8.53*	8.39*	8.12
	±0.66	±0.57	±0.76	±0.71

*: *p* < 0.05. None stimulation versus stimulation, mean ± standard deviation (cm/s)

Although there were no statistical significances, the LTV caused changes in each sub-phase. As a result, the elder’s weight shift ability increased in the TP when vibrations of the 180 Hz and 190 Hz were applied. Also, 250 Hz vibration increased weight shift though significant difference from the none stimulation didn’t appear.

### COM velocity along ML direction

There was no significant difference in the COM velocity along the ML direction in young adults (Table 5). The elderly showed a significant decrease in the COM velocity in the ML direction during the PP at 180 Hz and 190 Hz (Table 6).

**Table 5:** COM velocity in the ML direction in young adults

STS phase	None stimulation	180 Hz	190 Hz	250 Hz
PP	2.38	2.40	2.30	2.31
	±0.70	**±**0.70	**±**0.63	±0.55
RP	1.57	1.84	1.53	1.70
	±0.22	**±**0.26	±0.21	±0.27
SP	0.56	0.54	0.61	0.60
	±0.06	±0.08	±0.09	**±**0.12
TP	1.02	1.13	1.07	1.09
	±0.07	±0.13	±0.10	±0.10

*: *p* < 0.05. None stimulation versus stimulation, mean ± standard deviation (cm/s)

**Table 6:** COM velocity in the ML direction in the elderly

STS phase	None stimulation	180 Hz	190 Hz	250 Hz
PP	2.88	2.13*	2.09*	2.27
	±0.45	±0.51	±0.39	±0.48
RP	1.22	1.33	1.24	1.21
	±0.12	**±**0.21	±0.13	±0.14
SP	0.62	0.73	0.69	0.61
	±0.13	±0.13	±0.12	**±**0.11
TP	1.22	1.33	1.24	1.21
	±0.12	±0.21	±0.13	±0.14

*: *p* < 0.05. None stimulation versus stimulation, mean ± standard deviation (cm/s)

Even though decreased COM velocity appeared in the PP, COM velocity increased during the TP. This was attributed to increased COM velocity both during the RP and the SP. Therefore, the LTV can contribute positively to improve COM velocity in the ML direction.

### COM velocity along V direction

There was no significant difference in any of the STS movement phases except in the TP, where the velocity showed a significant increase (*p* < 0.05) at 180 Hz, 190 Hz, and 250 Hz (Table 7) for the young adults. The elderly showed a statistically significant increase (*p* < 0.05) in the velocity at the TP at 180 Hz and 190 Hz (Table 8).

**Table 7:** COM velocity along the V direction in young adults

STSphase	Nonestimulation	180 Hz	190 Hz	250 Hz
PP	14.17	12.11	13.07	11.97
	±1.47	±1.84	±2.57	±1.64
RP	27.68	27.96	27.19	28.16
	±2.23	**±**2.72	±2.76	±2.77
SP	0.51	0.41	0.39	0.31
	±0.46	±0.20	±0.19	**±**0.13
TP	9.66	10.07*	10.24*	10.13*
	±0.49	±0.55	±0.52	±0.61

*: *p* < 0.05. None stimulation versus stimulation, mean ± standard deviation (cm/s)

**Table 8:** COM velocity in the V direction in the elderly

STS phase	None stimulation	180 Hz	190 Hz	250 Hz
PP	14.06	14.91	14.98	15.83
	±2.93	±3.51	±3.51	±3.22
RP	21.77	19.75	20.58	20.01
	±2.53	**±**2.25	±1.91	±1.73
SP	0.25	0.30	0.26	0.25
	±0.14	±0.14	±0.13	**±**0.18
TP	8.72	9.24*	9.20*	9.12
	±0.56	±0.49	±0.50	±0.49

*: *p* < 0.05. None stimulation versus stimulation, mean ± standard deviation (cm/s)

Rising COM vertically is very important to perform and complete STS movement. It was shown that the LTV contributed to vertical transfer of the COM in both groups during the TP. In particular, although there were no statistical significances, the elderly presented increased COM velocity in the PP. This imply that the LTV affected trunk lifting via femoral abduction. Hence, further work is required in investigating biomechanical aspects of initial phases of STS movement.

### COP area

The COP area during the TP showed a statistically significant decrease (*p* < 0.05) at 180 Hz and 190 Hz in young adults (Table 9). In the elderly during the PP, it showed a significant increase at 180 Hz, 190 Hz and 250 Hz, but was significantly reduced during the RP (at 190 Hz). During the TP, it showed a statistically significant decrease (*p* < 0.05) at 190 Hz and 250 Hz (Table 10).

**Table 9:** COP area in young adults

STS phase	None stimulation	180 Hz	190 Hz	250 Hz
PP	21.772	22.587	21.783	24.081
	±3.492	±4.760	±4.690	±5.741
RP	12.417	11.190	11.648	12.343
	±3.574	**±**3.018	±2.150	±3.441
SP	4.131	5.006	3.671	5.599
	±0.685	**±**2.269	**±0.**869	**±**3.615
TP	144.004	126.870*	125.419*	130.643
	±14.402	±19.183	±18.978	±20.530

*: *p* < 0.05. None stimulation versus stimulation, mean ± standard deviation (cm^2^)

**Table 10:** COP area in the elderly

STS phase	None stimulation	180 Hz	190 Hz	250 Hz
PP	18.227	21.672*	20.056*	20.736*
	±0.957	±1.646	±1.392	±1.278
RP	16.778	14.257	12.412*	13.251
	±3.044	±2.880	±2.181	±2.875
SP	4.054	4.279	3.889	2.940
	±1.588	±1.523	±1.200	±0.965
TP	147.815	141.358	124.691*	121.524*
	±24.292	±24.252	±19.325	±20.363

*: *p* < 0.05. None stimulation versus stimulation, mean ± standard deviation (cm^2^)

In both groups, decreased postural sway appeared in all of frequencies. Particularly, in the elderly, postural sway of the RP and the SP decreased.

### COP path length

The COP path length for young adults showed a significant decrease (*p* < 0.05) at 190 Hz during the TP (Table 11). In the elderly, the TP showed a decrease (*p* < 0.05) at 190 Hz and 250 Hz (Table 12).

**Table 11:** COP path length in young adults

STS phase	None stimulation	180 Hz	190 Hz	250 Hz
PP	22.174	22.884	22.672	23.163
	±1.586	±2.184	±2.395	±2.666
RP	12.253	12.714	12.774	12.865
	±2.247	**±**2.467	±1.667	±1.928
SP	8.632	9.684	8.641	9.169
	±0.921	**±**3.041	**±**1.609	**±**1.800
TP	40.027	39.981	39.605*	40.018
	±2.308	**±**3.365	±3.034	±3.139

*: *p* < 0.05. None stimulation versus stimulation, mean ± standard deviation (cm)

**Table 12:** COP path length in the elderly

STS phase	None stimulation	180 Hz	190 Hz	250 Hz
PP	19.759	20.948	20.224	20.528
	±1.735	±1.573	±1.761	±1.853
RP	15.712	15.513	14.302	15.077
	±1.165	**±**2.399	±1.779	±2.579
SP	8.814	8.968	9.497	8.713
	±1.105	**±**2.404	**±**3.190	**±**3.096
TP	`42.422	41.181	38.796*	38.568*
	±3.010	**±**3.597	±3.612	±3.504

*: *p* < 0.05. None stimulation versus stimulation, mean ± standard deviation (cm)

There was a tendency for the COP path length to decrease in both groups, and the amount of decrease differs with frequencies. This means that the sway’s trajectory travel distance is reduced, and it will contribute to postural stability by reducing the deviation from the COM.

### COP RMS along AP direction

There was no significant difference in COP RMS along the AP direction in either group (Tables 13 and 14).

**Table 13:** COP RMS along AP direction in young adults

STS phase	None stimulation	180 Hz	190 Hz	250 Hz
PP	0.267	0.285	0.278	0.295
	±0.022	±0.036	±0.032	±0.053
RP	0.112	0.119	0.125	0.125
	±0.028	±0.027	±0.026	±0.026
SP	0.071	0.090	0.082	0.106
	±0.008	±0.034	±0.022	±0.056
TP	0.913	0.938	0.884	0.934
	±0.078	±0.086	±0.081	±0.095

*: *p* < 0.05. None stimulation versus stimulation, mean ± standard deviation (cm)

**Table 14:** COP RMS along AP direction in the elderly

STS phase	None stimulation	180 Hz	190 Hz	250 Hz
PP	0.232	0.244	0.236	0.241
	±0.025	±.024	±0.024	±0.023
RP	0.144	0.147	0.141	0.144
	±0.015	±0.022	±0.022	±0.031
SP	0.087	0.088	0.090	0.0843
	±0.014	±0.032	±0.040	±0.037
TP	0.839	0.831	0.844	0.866
	±0.105	±0.096	±0.093	±0.095

*: *p* < 0.05. None stimulation versus stimulation, mean ± standard deviation (cm)

### COP RMS along ML direction

The COP RMS along the ML direction in young adults showed a significant increase (*p* < 0.05) at 180 Hz and 190 Hz, but in the elderly, it showed a significant decrease (*p* < 0.05) at 190 Hz and 250 Hz (Tables 15 and 16).

**Table 15:** COP RMS along ML direction in young adults

STS phase	None stimulation	180 Hz	190 Hz	250 Hz
PP	0.047	0.059	0.057	0.056
	±0.006	±0.017	±0.017	±0.018
RP	0.083	0.085	0.080	0.085
	±0.014	±0.016	±0.012	±0.013
SP	0.077	0.075	0.067	0.077
	±0.012	±0.020	±0.013	±0.020
TP	0.175	0.199*	0.196*	0.189
	±0.014	±0.068	±0.071	±0.056

*: *p* < 0.05. None stimulation versus stimulation, mean ± standard deviation (cm)

**Table 16:** COP RMS along ML direction in the elderly

STS phase	None stimulation	180 Hz	190 Hz	250 Hz
PP	0.048	0.053	0.049	0.048
	±0.003	±0.006	±0.003	±0.004
RP	0.109	0.106	0.097	0.101
	±0.012	**±**0**.**026	±0.013	±0.018
SP	0.060	0.063	0.067	0.061
	±0.009	±0.013	±0.015	±0.016
TP	0.203	0.202	0.171*	0.170*
	±0.025	**±**0**.**043	±0.022	±0.021

*: *p* < 0.05. None stimulation versus stimulation, mean ± standard deviation (cm)

In the COP RMS in the ML direction, the effect of the LTV was different between the two groups. For the young adults postural sway in the ML direction increased, which is a negative effect that can cause postural instability, however it decreased for the elderly, which can improve postural perturbation. This means that there is a possibility that different effects can occur depending on neurophysiological state of the body. Hence, not only individual person’s response but also physiological function state must also be considered.

### Time durations

In young adults, the time duration of the SP was reduced (*p* < 0.05) at 180 Hz, 190 Hz, and 250 Hz. The TP time duration showed a reduction (*p* < 0.05) at 180 Hz, 190 Hz, and 250 Hz (Table 17). In the elderly, the SP time duration showed a reduction (*p* < 0.05) at 180 Hz, and the TP time duration showed a reduction (*p* < 0.05) at 180 Hz, 190 Hz, and 250 Hz (Table 18).

**Table 17:** Time duration in young adults

STS phase	None stimulation	180 Hz	190 Hz	250 Hz
PP	0.2888	0.3067	0.3173	0.2851
	±0.0540	±0.0879	±0.0752	±0.0662
RP	0.9607	1.0240	1.0578	1.0283*
	**±**0.0748	±0.1206	±0.1323	±0.1109
SP	1.9149	1.7471*	1.6276*	1.7382*
	±0.0987	±0.1761	±0.1823	±0.1436
TP	3.1884	3.0697*	3.0282*	3.0409*
	±0.0629	±0.0581	±0.0523	±0.0716

*: *p* < 0.05. None stimulation versus stimulation, mean ± standard deviation (s)

**Table 18:** Time duration in the elderly

STS phase	None stimulation	180 Hz	190 Hz	250 Hz
PP	0.2715	0.2875	0.2544	0.2546
	±0.0246	±0.0435	±0.0394	±0.0462
RP	1.2172	1.3070	1.2549	1.2966
	**±**0.1239	**±**0.1181	±0.0765	**±**0.0910
SP	1.8684	1.5499*	1.6343	1.6263
	±0.2195	±0.1810	±0.1502	±0.1298
TP	3.3364	3.1444*	3.1436*	3.1775*
	±0.1414	±0.0987	±0.0945	±0.0947

*: *p* < 0.05. None stimulation versus stimulation, mean ± standard deviation (s)

In both groups, the time required to complete the STS movement was reduced. This decrease is attributed to the decrease in the time duration in the SP. In other words, it means that the time to stabilize the posture has been reduced, and this suggests that the LTV has an advantage in securing postural stability.

## Discussion

### General features of STS movement in the elderly

While performing the STS movement, compared with the young adults, higher COP area, COP path length, COP displacements in the AP and the ML directions, and time duration, and also lower COM velocities in the AP and the V directions appear in the elderly. These tendencies are especially prominent in the RP.

During the RP, the elderly experience higher COP path length. COP displacements in the AP and the ML directions and time duration of the RP. Lesser postural stability is experienced at a larger COP path length in any direction [17]. In addition, the overall greater sway in the ML direction shows that the elderly have less stability compared to young adults [18]. COM velocity in the AP, the ML and the V directions are lower than that of the young adults. This delayed motor responses means that they generate effort in order to take-off from the seat at a relatively slow pace. This explains the longer time spent during the STS movement in the elderly compared to the young adults. In addition, the elderly shift their body weight to relatively stronger limb for stable body support, resulting in increasing COP sway in the ML direction during the RP.

In the SP, even though the elderly exhibit lesser sway in the ML direction, they show greater sway in the AP direction. This could be because they use a lot of effort in trying to break the initial impulse from the RP. During the RP, head, arm and trunk (HAT) are flexing and then extending, also lower-limbs are continuously extending. These motions of segments contribute to an increase in postural sway.

In summary, elderly individuals generally have higher postural sways, lower COM shift ability and performance competence during the STS movement.

### Vibrations in young adults

Vibration did not have a significant effect on the COP and COM in young adults during the PP. Although time duration was reduced at 250 Hz, all other parameters remained the same during the RP. The SP showed time duration reductions at 180 Hz, 190 Hz, and 250 Hz. The total STS movement showed a different effect as a result of vibrations. At 180 Hz, the COP sway area decreased, the STS time duration decreased, and the COP displacement in the ML and the COM velocity in the V direction increases. At 190 Hz, the COP sway area, STS time duration, and COP path length decreased, COP displacements in the ML, COM velocities in the V and AP directions, and COM displacement increased. At 250 Hz, the COM displacement and COM along the V direction increased and the time duration reduced. The results show that vibration at 190 Hz has the highest effect on young adults during STS movement. Young adults use the momentum transfer strategy by generating sufficient horizontal momentum to move the COM [19] from the seating position to the base of support in a stable standing position. Young adults transfer momentum from the sitting to standing position faster with reduced foot sway during vibration. Vibration further improves the postural control system in young adults.

### Vibrations in the elderly

The elderly react differently when the LTV is applied during the STS movement. During the PP, there was an increase in COP sway area at 180 Hz, 190 Hz, and 250 Hz and a reduction in the COM velocity in the ML direction at 180 Hz and 190 Hz. This could be a result of corrections made in the trunk region by the LTV. The elderly first perform trunk flexion and push the body forward while reducing the COM velocity in the ML direction to overcome gravity before rising from the seat and standing erect [15]. This strategy known as trunk flexion that is used by elderly to ensure postural stability during STS movement [20] is improved by the LTV. In the RP, the COP sway area reduces only at 190 Hz. During the SP, statistically significant reduction in the time duration appears at 180 Hz, although both the COP sway area in the RP and the time duration in the SP tend to decrease for all frequencies*.* The TP provides a better understanding of their response to vibration. The total STS phase shows that after vibration is applied, the COP area sway at 190 Hz 250 Hz, COP path length 190 at Hz and 250 Hz, COP displacement in the ML direction at 190 Hz and 250 Hz, and the total time duration at 180 Hz, 190 Hz, and 250 Hz decreased. The COM velocity in the AP direction at 180 Hz and 190 Hz and in the V direction at 180 Hz and 190 Hz increased, but the COM displacement at 190 and 250 Hz decreased. Through these results, it was found that vibration of 190 Hz is the most effective in ensuring postural stability in elderly.

In summary, the LTV improves postural instability by decreasing the COP sways while performing the STS movement. Furthermore*,* the STS performance is improved in the elderly, to resemble that of the young adults, by increasing the COM velocity in the V direction and decreasing the time duration. Finally, the COM velocities in the AP and V directions increased, but the COM displacement decreased as a result of improvement in trunk flexion strategy by the LTV. Thus, further study is required to investigate aspects of kinematics and kinetics when STS movement is conducted because the LTV affects the trunk flexion strategy and the extent of this effect changes depending on frequency.

## Conclusion

The aim of this study was to investigate the effect of LTV on postural control in the elderly during STS movement. To do this, changes in the COP and COM when the LTV was applied were examined for both young adults and the elderly, and the main findings were as follows:

First, although the elderly have different strategies to prevent fall and the aging effects on postural control compared to younger persons during STS movement, the elderly show higher postural sway and lower weight shift ability.

Second, the LTV affected both postural sway and weight shift ability in the elderly, and the extent of the changes in postural sway and weight shift depends on frequency of the LTV. Especially, the 190 Hz frequency was recognized as the best frequency when applied throughout the whole phase of STS movement to cause motor responses in older persons. The trunk flexion strategy is improved by the LTV and the elderly exhibit lesser COP sways in all directions. In addition, STS performance is improved by reducing the time spent during the entire STS and increasing the COM velocity.

These findings mean that the LTV has a positive contribution to improve postural stability and performance in the elders’ STS movement, and suggest that individual person’s response should be considered when the LTV is applied because there exists an extent difference dependent on properties of the LTV. Finally, the results of present study can provide clinical information to improve weakened postural control and can be used to design movement assisting aids.

## Data Availability

The datasets used and/or analyzed during the current study are available from the corresponding author on reasonable request.

## Abbreviations

COM: Center of mass
COP: Center of pressure
STS: Sit-to-stand
AP: Anterior-posterior ML Medio-lateral
V: Vertical
LTV: Local tendon vibration
PP: Preparation phase
RP: Rising phase
SP: Stabilization phase
TP: Total phase
